# 

**Published:** 1857-06

**Authors:** 


### ELEVENTH VOLUME

OF THE

### DENTAL REGISTER OF THE WEST.

EDITED BY

**J. TAFT and G. WATT.**

TERMS: - - - - - - - - $3 PER ANNUM, IN ADVANCE.

### Advertisements Inserted as Follows:

| | |
|---|---|
| One page, per annum | $25 00 |
| One-half page, per annum | 15 00 |
| Quarter page, per annum | 10 00 |

Shorter periods than one year, by special arrangement.

☞Contributions to the pages of the *Register* respectfully solicited.

Exchanges and correspondents will please address, " Dental Register," or " Taft & Watt, Cincinnati, Ohio."

